# Adult ADHD and emerging models of maladaptive personality: a meta-analytic review

**DOI:** 10.1186/s12888-021-03284-1

**Published:** 2021-06-01

**Authors:** Peter Jacobsson, Christopher J. Hopwood, Bo Söderpalm, Thomas Nilsson

**Affiliations:** 1 The Sahlgrenska Academy at University of Gothenburg, Gothenburg, Sweden; 2 Psychiatry Halland, Region Halland, Sweden; 3 University of California, Davis, USA; 4 Department of Forensic Psychiatry, National Board of Forensic Medicine, The Sahlgrenska Academy at University of Gothenburg, Gothenburg, Sweden

**Keywords:** Personality, Personality disorder, ADHD, Personality traits, Clinical assessment, Diagnosis

## Abstract

**Background:**

ADHD is a highly consequential disorder that is estimated to affect 2.5% of the adult population. Emerging models of psychopathology posit that disorders like ADHD can be usefully situated within general models of individual differences in personality, such as those recently implemented in the DSM and ICD for the diagnosis of personality disorder. Previous research and systematic reviews have linked adult ADHD to the personality traits Conscientious Inhibition and Negative Emotionality. However, there have been some inconsistencies in the literature and research embedding ADHD-personality connections in the DSM-5 and ICD-11 personality disorder models has been limited. The goal of this paper was to systematically review associations between adult ADHD and personality traits, organized within a maladaptive five factor framework.

**Method:**

A comprehensive literature search yielded 13 papers whose effects were meta-analyzed.

**Results:**

Results supported associations between ADHD and low Conscientious Inhibition and high Negative Emotionality. However, interesting patterns of variability were observed, potentially related to issues such as instrumentation and facet variation.

**Conclusion:**

Results support the clinical application of personality assessment for suggesting risk for ADHD symptoms, and point to important directions for further research.

Attention Deficit Hyperactivity Disorder (ADHD) is a prevalent psychiatric disorder [1–3]. Although it is commonly conceptualized as a neurodevelopmental condition, it also includes features that resemble basic personality traits, such as Neuroticism and Impulsivity [4, 5]. Similar to broad personality traits, ADHD tends to co-occur with a wide range of other disorders, including mood disorders, anxiety disorders, personality disorders and substance disorders [6–9]. Moreover, deficits in personality traits such as emotion-regulation, distractibility, irresponsibility, risk-taking and impulsivity are thought to be at the core of ADHD symptoms [10, 11].

Most research on personality and psychopathology organizes individual differences in the form of the Five Factor Model [4], which includes the traits Neuroticism, Extraversion, Openness, Agreeableness, and Conscientiousness. Notably, several comparable models have gained attention in mental health research and practice recently, including the Research Domain Criteria (RDoC) [12]; the Hierarchical Taxonomy of Psychopathology (HiTOP) [13]; the Alternative Model of Personality Disorder (AMPD) [14]; and the ICD-11 proposal for personality disorder [15]. All of these models have in common the proposal that a few broad traits underlie functioning in a wide range of areas, including dysfunction related to inattention, impulsivity and hyperactivity. Associations between personality and psychopathology are particularly strong when maladaptive trait measures, or instruments that focus on personality-related problems, are used [16].

Several previous reviews have documented associations between FFM personality traits and ADHD [17–19]. These reviews indicate that ADHD as a unified construct is most consistently related to low Conscientiousness, low Agreeableness, and high Neuroticism. This research also suggests some specificity in associations between inattentive and hyperactive/impulsive aspects of ADHD and certain traits [20, 21]. Specifically, Inattention is positively correlated to Neuroticism and negatively to Conscientiousness; whereas Hyperactivity/Impulsivity relates negatively to Agreeableness and positively to Extraversion [17]. As such, there is considerable potential in using basic traits like those of the FFM, RDoC, HiTOP, AMPD, and ICD-11 to understand how individuals diagnosed with ADHD differ from individuals with other diagnoses in terms of underlying personality traits. The goal of the present study was to use meta-analysis to summarize associations between adult ADHD and create a bridge from non-clinical to maladaptive personality traits.

## The integrated five-factor model

The FFM consists of the following five factors: *Neuroticism* which refers to the extent of negative emotions, i.e., sadness, fear, hostility, and emotional lability that the individual experiences [22]. Individuals high in this domain may be at risk for many different psychiatric and physical disorders, high comorbidity, lower quality of life, shorter lifespan and more extensive use of health care [23]. *Extraversion* concerns how outgoing or talkative the individual is in most situations. Core traits are sociability, assertiveness, positive affect, and activity level [22]. Low Extraversion, or introversion, may include social withdrawal, social detachment, intimacy avoidance, restricted affectivity and anhedonia [24–26] and extremely high Extraversion may represent personality pathology in sexual promiscuity, emotional intrusiveness, excessive self-disclosure and thrill-seeking behavior [27]. *Openness to experience* describes the depth and breadth of an individual’s intellectual, artistic, and experiential life, with key facets such as aesthetic sensitivity, intellectual interests, and imagination [22]. Although open individuals generally tend to have greater psychological well-being, maladaptive Openness can be found in distinguishing between major depression and bipolar disorder as well as different variants of schizotypy [28]. *Agreeableness* concerns the extent of the individual’s motivation for prosocial behavior and pleasant interpersonal relationships. Important traits are compassion, trust, and politeness [22]. Individuals low in Agreeableness tend to be critical, skeptical, try to push limits, express hostility and being condescending [28] . Agreeableness is also inversely linked to psychopathy and aggressive behavior [29, 30]. Maladaptive variants of extreme Agreeableness can result in gullibility, submissiveness, clinging, subservience, servility etc. [28]. *Conscientiousness* refers to the individual’s level of organization, ability to complete tasks, and persistence in achieving long-term goals. Key concepts are orderliness, self-discipline, and reliability [22]. However, both high and low Conscientiousness is associated with decreased functioning. Low Conscientiousness is characterized by disinhibition, irresponsibility, negligence, and rashness [31, 32]; and inflexible high Conscientiousness can result in perfectionism, fastidiousness, punctiliousness, workaholism and other facets of compulsivity [33–35].

Research has shown that multiple models of both normal-range and maladaptive personality traits can be integrated into the structure of the FFM [36–38]. For instance, the trait Neuroticism is empirically and conceptually similar to the trait Negative Affectivity in the AMPD and ICD-11, Emotion Dysregulation in Livesley’s Dimensional Assessment of Personality Pathology (DAPP [39];, and the traits Harm Avoidance and (low) Self-Transcendence in Cloninger’s Temperament and Character Inventory (TCI [40];. Similarly, FFM Conscientiousness is similar to Disinhibition in the AMPD/ICD-11, Compulsivity on the DAPP-BQ, and low Novelty Seeking and Persistence in the TCI. As such, in this study, we will conceptualize personality traits in terms of an Integrative Five Factor Model (IFFM) inclusive of instruments that are explicitly conceptualized as measures of the FFM, as well as those, such as the DAPP and TCI, that have commensurate scales (See Table 1). We will use domain labels that integrate normal-range and maladaptive aspects of each trait [37]: Negative Emotionality, Positive Emotionality, Openness, Conscientious Inhibition, and Agreeable Inhibition.

**Table 1:** A translation of how personality traits from different assessment instruments are related to the integrated five-factor model traits

The Integrated Five-Factor Model^a^	Five-Factor Model	DAPP-BQ	Cloninger TCI	AMPD/ICD-11^b^
*Negative Emotionality*	Neuroticism	Emotional dysregulation	Harm avoidance; Self-directedness (−)	Negative affectivity
*Positive Emotionality*	Extraversion	Inhibitedness (−)	Harm avoidance (−); Reward dependence	Detachment (−)
*Openness*	Openness to experience		Self-transcendence	Psychoticism (AMPD only)
*Conscientious Inhibition*	Conscientiousness	Compulsivity	Novelty seeking (−); Persistence	Disinhibition (AMPD) (−); Anankastia (ICD-11)
*Agreeable Inhibition*	Agreeableness	Dissocial behavior (−)	Cooperativeness	Antagonism (AMPD) (−); Dissociality (ICD-11) (−)

^a^Adapted from Markon et al (2005) [37] and Gomez & Corr (2014). Note: Negative sign indicates that the scale loaded negatively

^b^Emerging personality models AMPD and ICD-11 factors are not represented in included studies of the current review

## Conceptualizing ADHD within an integrative individual differences framework

Conceptualizing ADHD as a dimension or set of dimensions that can be placed within a general, evidence-based model of maladaptive personality traits has a number of potential clinical advantages [41–44]. For instance, this model may help provide a principled and efficient means for conceptualizing heterogeneity in the presentation of individuals with ADHD diagnosis. Specifically, previous research suggests that Negative Emotionality and low Conscientious Inhibition are most strongly related to inattention, whereas Agreeable Inhibition and Positive Emotionality maybe more strongly related to hyperactivity and impulsivity [17, 45–47]. Evidence-based models of individual differences also help explain comorbidity. Insofar as many disorders share underlying propensities for high Negative emotionality and low Conscientious and Agreeable Inhibition [48], these disorders should be expected to co-occur with inattentive, impulsive and hyperactive symptoms. Conceptualizing ADHD within a multidimensional personality model that can help provide explanations and order to patterns of heterogeneity and comorbidity may thus pave the way for more targeted treatments and research. As such, individual differences models such as the IFFM have the potential to improve the efficiency, validity, and utility of ADHD diagnoses [43].

Four previous studies summarize associations between personality traits and adult ADHD [17–19, 47]. This study provides additional information not included in those studies in the following ways. First, this unlike some reviews [18], uses formal meta-analytic procedures to quantify associations between personality traits and ADHD. Second, six not previously reviewed studies are included in the current meta-analytic review [46, 49–53]. Third, whereas the cited earlier reviews have included both child and adult studies with varying approaches to the assessment of ADHD [17–19, 47], the present review has only included papers with adult individuals and excluded studies without a clear description of a formal ADHD diagnosis using a stringent clinical assessment. Fourth, this study conceptualizes personality using various models that can be integrated within an IFFM framework.

In summary, the aim of this study was to quantify associations between ADHD and the traits of an Integrated FFM to better understand how ADHD fits into evidence-based models of personality and psychopathology, and ultimately enable improved clinical practice and research. Given the existing body of research in adult populations, we focused on traits from the FFM, DAPP, HEXACO [54], and TCI. Given that relatively few studies have distinguished between ADHD subtypes, we conceptualized ADHD as unitary construct.

## Method

**Fig. 1:**
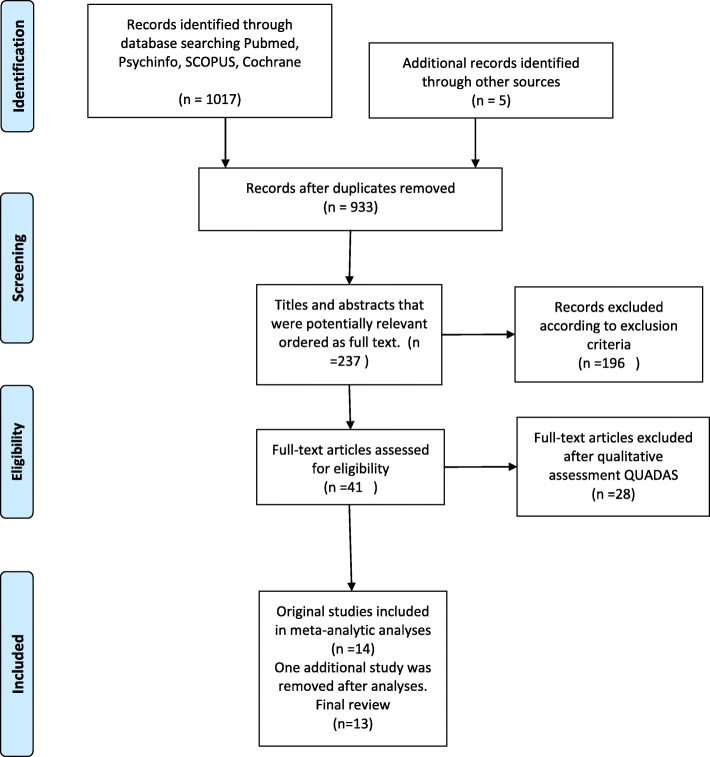
PRISMA 2009 Flow Diagram

Literature searches were conducted in November 2019 via Cochrane, PubMed, PsychInfo and SCOPUS using the following keywords: (ADHD OR ADD OR ADDH OR Attention Deficit Disorder with Hyperactivity) AND (personality traits OR personality dimensions OR NEO OR five-factor model OR FFM OR BFI OR Temperament and Character Inventory OR TCI). The reference sections of each article retrieved was searched manually for any potentially relevant studies. Included studies were a) written in English; b) published after January 1, 2000; c) contained adult cases with a diagnosis of ADHD according to DSM-IV or DSM-5; d) used validated measures to assess adult ADHD; e) used established dimensional personality measures (FFM, DAPP, HEXACO, or TCI); and f) presented results in a format that allowed for straightforward conversion to the FFM i.e. into the Integrated Five-Factor Model (IFFM [37]; (Fig. 1).

The first author screened titles and abstracts and excluded papers that did not meet inclusion criteria. The remaining articles were assessed, discussed, and consented upon by the first author and two of the coauthors (TN and BS). This process resulted in 14 papers that sampled adult patients with ADHD [17, 46, 49–53, 55–61]. Six studies used an explicit FFM measure [17, 46, 49, 50, 52, 53], one used the HEXACO [61], six used the Temperament and Character Inventory (TCI [55–60];, and Jacob et al. [51] included both FFM and TCI measures. A study by Koerting et al. [52] used measures from the DAPP-BQ in addition to an FFM instrument. As explained above, and illustrated in Table 1, traits from these measures were categorized as falling within the IFFM domains of *Negative Emotionality*, *Positive Emotionality*, *Openness*, *Conscientious Inhibition*, or *Agreeable Inhibition*. Within the IFFM framework, the DAPP-BQ traits *Emotional dysregulation* and *Compulsivity* relate to *Negative Emotionality and Conscientious Inhibition,* respectively; *Inhibitedness* loads negatively on *Positive Emotionality;* and *Dissocial behavior* has a negative association with *Agreeable Inhibition*. No specific DAPP-BQ trait is associated with Openness in the IFFM framework. The TCI traits *Harm avoidance*, *Reward dependence*, *Self-transcendence*, *Persistence*, and *Cooperativeness* are associated with the respective IFFM domains *Negative Emotionality*, *Positive Emotionality*, *Openness*, *Conscientious Inhibition*, and *Agreeable Inhibition*. *Self-directedness* loads negatively on *Negative Emotionality*, *Harm avoidance* is negatively related to *Positive Emotionality*, and *Novelty seeking* is negatively related to *Conscientious Inhibition*.

All formal ADHD diagnoses were based on the DSM criteria, although different instruments were used to assess current and historical symptoms and impairments. All data included in this analysis examined group differences as they relate to personality traits. Control groups included community samples, non-clinical parents of children with ADHD, blood donors, clinical groups without ADHD, and population norms extracted from manuals (Table 2). In five of the papers [17, 46, 51, 58, 62] correlations with the ADHD symptom clusters: Combined (C), Inattentive (I), Hyperactive/Impulsive (HI), were described. Only the Jacob [51] 2016 study presented individual data according to specific subtype of ADHD. Thus, results on individual ADHD type or presentation could not be aggregated and analyzed separately. However, indicative heuristic patterns are presented and discussed.

**Table 2:** A compilation of included studies presenting study characteristics, data about personality scales (*M, SD*,) for ADHD and control subjects, and correlations between personality traits and ADHD

Author	ADHD N	Male (%)	Age ***M (SD)***	Clinical recruitment	Control N	Male (%)	Age ***M (SD)***	Control Group	Personality scale	ADHD ***M (SD)***	Control ***M (SD)***	Correlation trait - ADHD dx
He & Antschel (2019)	184	51	36.3 (10.8)	Psychiatric clinics	118	46	29.8 (8.7)	Community	TCI Harm Avoidance	60,0 (13,5)	52,78 (9,9)	.29
									TCI Self-directedness	61,4 (13,9)	80,3 (9,3)	−.62
									TCI Reward Dependence	67,2 (11,2)	72,0 (9,4)	−.23
									TCI Self-transcendence	52,4 (16,5)	48,8 (16,3)	.11
									TCI Novelty seeking	64,0 (9,7)	53,0 (7,2)	.54
									TCI Persistence	63,3 (11,8)	72,9 (9,9)	−.40
									TCI Cooperativeness	80,3 (9,2)	74,5 (11,0)	−.27
Wallace (2016) [49]	217	56	23.6 (4.7)	Psychiatric clinics and advertisements	114	50	22.2 (4.7)	Community	NEO FFI Neuroticism	28,5 (7,9)	21,50 (7,8)	.40
									NEO-FFI Extraversion	28,8 (7,7)	29,5 (7,4)	−.05
									NEO-FFI Openness to experience	33,6 (7,1)	31,9 (6,5)	.12
									NEO-FFI Conscientiousness	16,1 (4,8)	20,4 (4,6)	−.42
									NEO-FFI Agreeableness	31 (6,9)	32,9 (6,6)	−.14
Di Nicola (2014) [50]	16	No information	No information	Affective disorders unit	102	38^a^	47.4 (13.2)	BD	NEO-PI-R Neuroticism	140,5 (23,5)	112,0 (21,4)	.54
	8	No information	No information	Affective disorders unit	106	40^a^	46.1 (14.3)	MDD		133,4 (22,9)	111,3 (22,8)	.44
								BD	NEO-PI-R Extraversion	93,4 (25,0)	94,8 (21,5)	−.03;
								MDD		82,3 (20,3)	93,1 (20,9)	−.25
								BD	NEO-PI-R Openness	104,7 (21,3)	105,8 (20,7)	−.03
								MDD		107,4 (21,1)	101,4 (14,5)	.16
								BD	NEO-PI-R Conscientiousness	88,7 (17,9)	106,0 (21,0)	−.41
								MDD		94,4 (22,7)	108,6 (21,8)	−.30
								BD	NEO-PI-R Agreeableness	118,5 (18,3)	119,0 (18,9)	−.01
								MDD		124,0 (15,6)	119,2 (15,4)	.15
Faraone et al. (2009) [56]	127	53	36.1 (10.8)	Psychiatric clinics and advertisements	123	46	29.9 (9.0)	Community	TCI Harm Avoidance	59,5 (13,5)	52,7 (9,95)	.26,
									TCI Self-directedness	61,3 (13,5)	80,2 (9,4)	−.57
									TCI Reward Dependence	67,4 (10,7)	72,0 (9,5)	−.20
									TCI Self-transcendence	52,4 (16,8)	48,6 (16,2)	.12
									TCI Novelty seeking	64,2 (10,6)	53,2 (7,2)	.53
									TCI Persistence	63,1 (12,2)	72,9 (10,0)	−.33
									TCI Cooperativeness	75,6 (10,0)	80,2 (9,2)	−.23
Jacob et al. (2016) [51]	Male 441	50	34.5 (10.2)	Psychiatric clinic	80	No information	No information	Reference values male	TPQ Harm Avoidance	18,2 (7,1)	14,7 (6,4)	.25
	Female 440	50	34.5 (10.2)	Psychiatric clinic	80	No information	No information	Reference values female		20,9 (6,7)	16,3 (6)	.34
									TPQ Reward Dependence	16,1 (4,5)	14,8 (4,7)	.14
										18,6 (4,2)	17,5 (4,7)	.12
									TPQ Novelty seeking	19,5 (5,7)	12,8 (5,8)	.50
										19 (5,9)	15,1 (4,6)	.34
	Male 426		34.5 (10.2) Total	Psychiatric clinic	4219	No information	No information	Reference values male	NEO-PI-R Neuroticism	108,7 (24,6)	84,2 (22,5)	.46
	Female 432		34.5 (10.2) Total	Psychiatric clinic	7505	No information	No information	Reference values female		122,6 (25,1)	95 (23,3)	.50
									NEO-PI-R Extraversion	100,1 (21,6)	109,6 (20,4)	−.22
										100,3 (23,7)	111 (19,5)	−.24
									NEO-PI-R Openness	109,4 (19,1)	120,4 (20,1)	−.27
										114,2 (21,2)	125,7 (18,6)	−.28
									NEO-PI-R Conscientiousness	92,3 (21,2)	114,1 (20,7)	−.46
										89,9 (22,8)	113,8 (19,7)	−.49
									NEO-PI-R Agreeableness	107,7 (16,9)	108,6 (17,3)	−.03
										116 (16,1)	114,9 (16,4)	.03
Koerting (2016) [52]	30	43	33.5 (8.8)	Psychiatric clinic	30	43	28.2 (7.0)	Community	NEO-PI-R Neuroticism;	2,4 (0,4)	1,8 (0,4)	.58
									NEO-PI-R Extraversion	2,1 (0,4)	2,3 (0,3)	−0.46
									NEO-PI-R Openness	2,5 (0,4)	2,5 (0,3)	−0.30
									NEO-PI-R Conscientiousness	1,6 (0,5)	2,5 (0,4)	−0.01
									NEO-PI-R Agreeableness	2,3 (0,3)	2,4 (0,3)	0.11
									DAPP-BQ Emotional Dysregulation	2,7 (0,5)	2,1 (0,4)	.52
									DAPP-BQ Inhibitedness	2,5 (0,4)	2,0 (0,3)	.56
									DAPP-BQ Compulsivity	2,6 (0,7)	3,0 (0,6)	−.31
									DAPP-BQ DissocialBehavior	2,5 (0,4)	2,3 (0,4)	.32
Nigg et al. (2002) [17]	88	38	21.6 (3.9)	University disability office and advertisements	26	No information	No information	Community	NEO-FFI Neuroticism	26,4 (8,7)	19,2 (7,3)	.41
									NEO-FFI Extraversion	32 (7,2)	31,3 (4,7)	.06
									NEO-FFI Openness to experience	31,6 (5,8)	30,7 (5,9)	.08
									NEO-FFI Conscientiousness	22,3 (7,6)	34,6 (5,2)	−.69
									NEO-FFI Agreeableness	29,7 (6)	33,3 (5,9)	−.29
Knouse (2013) [46]	117	50	42.8 (11.0)	Hospital	1539	No information	No information	Reference values	NEO FFI Neuroticism	26,3 (8,2)	19,1 (7,7)	.41
									NEO-PI-R Extraversion	28,5 (6,4)	27,7 (5,8)	.07
									NEO-PI-R Openness	31,3 (6,5)	27,0 (5,8)	.33
									NEO-PI-R Conscientiousness	19,7 (6,7)	34,6 (5,9)	−.76
									NEO-PI-R Agreeableness	31,7 (6,5)	32,8 (5,0)	−.10
Sizoo et al. (2009) [57]	53	68	31.4 (9.9)	Center developmental disorders	657	No information	No information	Expected population norms (T-score)	VTCI Harm Avoidance	55,2 (11,4)	50 (10)	.24
									VTCI Self-directedness	34,8 (14,8)	50,0 (10,0)	−.52
									VTCI Reward Dependence	50,3 (10,9)	50,0 (10,0)	.14
									VTCI Self-transcendence	53,7 (10,8)	50,0 (10,0)	.18
									VTCI Novelty seeking	61,3 (9,6)	50,0 (10,0)	.50
									VTCI Persistence	50,5 (10,7)	50,0 (10,0)	.02
									VTCI Cooperativeness	46,1 (13,4)	50,0 (10,0)	.16
Steinhausen et al (2013) [53]	30	No information	No information	Parents with ADHD of children with ADHD	34	50	44.7 (5.4)	Parents of children with ADHD	NEO-FFI Neuroticism	2,2 (0,8)	1,5 (0,6)	.44
									NEO-FFI Extraversion	2,2 (0,7)	2,4 (0,5)	−0.16
									NEO-FFI Openness to experience	2,3 (0,5)	2,4 (0,4)	−0.11
									NEO-FFI Conscientiousness	2,4 (0,6)	2,9 (0,5)	−0.41
									NEO-FFI Agreeableness	2,5 (0,5)	2,6 (0,4)	−0.11
Perroud (2016) [58]	119	69	37.3 (11.0)	ADHD center	403	63	45.5 (12.7)	Blood donors	TCI Harm Avoidance	18,3 (8,1)	12,7 (6,2)	.36
									TCI Self-directedness	21,9 (8,5)	34,5 (5,8)	−.65
									TCI Reward Dependence	14,2 (4,0)	14,6 (3,8)	−.05
									TCI Self-transcendence	15,3 (6,8)	13,5 (6,3)	.14
									TCI Novelty seeking	23 (5,9)	18,8 (5,6)	.34
									TCI Persistence	4,8 (2,0)	4,8 (2,0)	.00
									TCI Cooperativeness	30,5 (7,4)	33,5 (5)	−.23
Sizoo et al. (2015) [59]	53	66	32.1 (11.4)	Center developmental disorders	399	No information	No information	Expected population norms (T-scores)	VTCI Harm Avoidance	55,7 (11,4)	50,0 (10,0)	.26
									VTCI Self-directedness	34,4 (14,7)	50,0 (10,0)	−.53
									VTCI Reward Dependence	50 (10,7)	50,0 (10,0)	.00
									VTCI Self-transcendence	53,8 (10,8)	50,0 (10,0)	.18
									VTCI Novelty seeking	60,9 (9,7)	50,0 (10,0)	.48
									VTCI Persistence	50,6 (10,7)	50,0 (10,0)	.03
									VTCI Cooperativeness	45,9 (13,2)	50,0 (10,0)	−.17
Anckarsater et al. (2006)	100	No information	No information		1300	No information	No information	Expected population norms (T-scores)	TCI Harm Avoidance	63,4 (12,1)	50,0 (10,0)	.51
									TCI Self-directedness	29 (12,5)	50,0 (10,0)	−.68
									TCI Reward Dependence	46,2 (12,5)	50,0 (10,0)	−.16
									TCI Self-transcendence	55,1 (12,9)	50,0 (10,0)	.22
									TCI Novelty seeking	59,2 (10,9)	50,0 (10,0)	.40
									TCI Persistence	50,2 (10,1)	50,0 (10,0)	.01
									TCI Cooperativeness	35,2 (17,5)	50,0 (10,0)	−.46

*Note*: *TCI* Temperament and Character Inventory, *NEO FFI* NEO Five Factor Inventory, *NEO-PI-R* The Revised NEO Personality Inventory, *TPQ* Tridimensional Personality Questionnaire, *DAPP-BQ* Dimensional Assessment of Personality Pathology, Brief Questionnaire, *VTCI* The brief Dutch version of the Temperament and Character Inventory, *BD* Bipolar Disorder, *MDD* Major Depressive Disorder

^a^Total number including ADHD

Meta-analysis was used to aggregate associations of personality traits and ADHD in Meta-Essentials [63, 64]. Group differences were converted to the Cohen’s d metric, weighted by sample size, and analyzed separately for each personality dimension. Effect sizes, 95% confidence intervals and prediction intervals with a random effects model were calculated and illustrated in forest plots. Heterogeneity of effects was also calculated to estimate the robustness of results [65]. I^2^ was used to evaluate the heterogeneity of mean effect sizes. An I^2^ value above 50% can be interpreted as meaningful heterogeneity around the mean effect size [66].

### Quality assessment of papers

The 14 papers were ranked for scientific quality according to an adaptation of QUADAS [67]. QUADAS was developed as a tool to systematically assess scientific papers that concern diagnostic tests. The following QUADAS items: a) design, b) sample size, c) relevant population, d) assessment method, e) relevant assessment instrument, f) sources of recruited cases, g) blinding, h) conflict of interest, and i) drop-out, were assessed by the first author (PJ) and two co-authors (TN and BS). The papers were first assessed independently and then discussed to reach consensus. Each aspect was scored from 0 to 2: unsatisfactory = 0; fair = 1; good = 2. The scores for the aspects were summarized for each paper to generate total scores between 0 and 18. The 14 papers ranged from 5 to 13. The paper with lowest score [5] was removed after quality assessment and subsequent preliminary analyses due to deviating both in quality assessment and being identified as an outlier in subsequent analyses, thus resulting in 13 papers that were included in the final analysis. The excluded paper did not meet the quality criteria (unsatisfactory = 0) for five of the nine assessed criteria: small sample, limited number of sources from which participants were recruited, no blinding, no description of conflict of interest, no description of drop-out. High drop-out rate or not specifying drop-out and insufficient blinding were the most common quality deficits in the other papers. Assessment methods and relevant populations were generally study strengths. The range of the included papers was 7 to 13 (median 10).

## Results

Thirteen original articles consisting of 2023 unique adult subjects diagnosed with ADHD and 16,835 control participants, were included in this meta-analysis. Mean age ranged from 21.6 to 42.8 years; 54% of the included population identified as male. Most of the clinical cases of ADHD were recruited through psychiatric clinics, either general or specialized centers (see Table 2). A few clinical cases were recruited through advertisements.

**Fig. 2:**
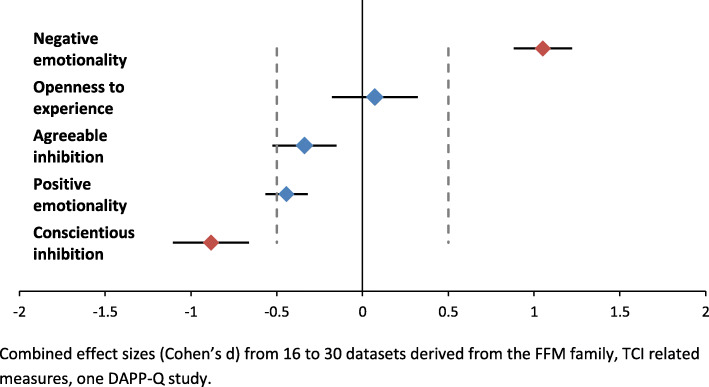
Personality traits in ADHD

The meta-analytic analyses used in this review indicated a range of combined effect sizes from d = 0.15 (negligible) for Openness to d = 1.11 (large) for Negative Emotionality [68, 69] (Fig. 2). The effect size for Conscientious Inhibition was also large (d = − 0.89). Effect sizes for Agreeable Inhibition and Positive Emotionality were more modest, d = − 0.39 and − 0.43, respectively. All associations with the exception of Openness were significant. *Negative Emotionality* and *Conscientious Inhibition* are thus the personality dimensions that were consistently and most strongly elevated in the ADHD samples in this review.

**Fig. 3:**
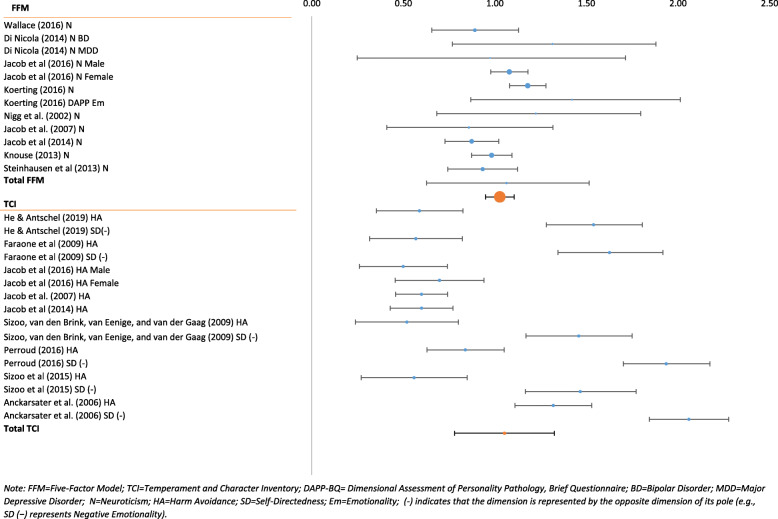
Negative Emotionality in ADHD

**Fig. 4:**
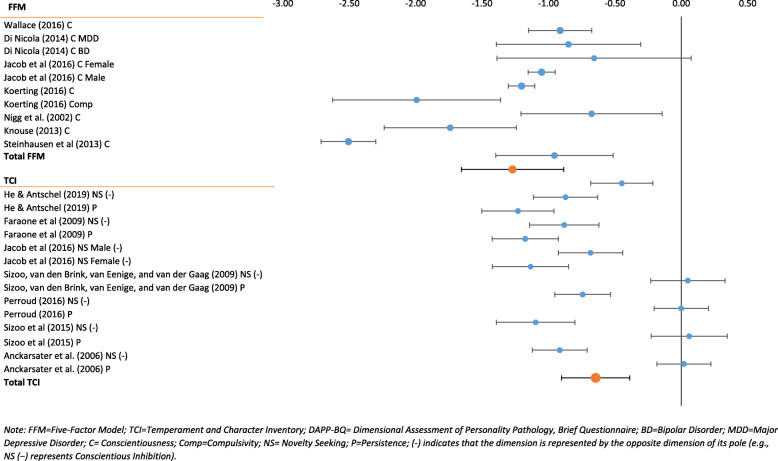
Conscientious Inhibition in ADHD

Even though the combined effect sizes for Negative Emotionality and Conscientious Inhibition were large, there was significant variability in the ADHD populations within these personality domains. Effect sizes for Negative Emotionality ranged from d = 0.50 (medium to large) to d = 2.06 (very large) (Fig. 3). An even greater range was observed for Conscientious Inhibition: d = 0.06 (negligible) for the Cloninger TCI facet Persistence to d = − 2.51 (very large) for the Five-Factor domain Conscientiousness (Fig. 4). Combined confidence intervals (CI) and prediction intervals (PI) were largest for Conscientious Inhibition (CI = -1.15 to − 0.63; PI = − 2.09 to 0.30) and smallest for Positive Emotionality (CI = -0.57 to − 0.28; PI = − 1.23 to 0.38).

The I^2^ is a measure for the proportion of observed variance that reflects real differences in effect size. It is expressed as a percentage with a range from 0 to 100% [70]. All effect sizes for the various personality domains in the ADHD samples ranged from 85 to 97%. Heterogeneity decreased when FFM measures were used exclusively. The I^2^ for FFM Negative Emotionality was 47% and I^2^ for FFM Agreeable Inhibition was 64%.

## Discussion

The results of this systematic, quantitative review suggest a robust connection between adult ADHD and the IFFM personality domains *Negative Emotionality* and *low Conscientious Inhibition*. *Positive Emotionality* and *Agreeable Inhibition* have somewhat smaller, although significant, associations with adult ADHD. These results are consistent with the review from Gomez et al. [47], with the exception that Gomez et al. observed a larger effect size for Agreeable Inhibition (d = −.64) than we did (d = −.39).

Low *Conscientious Inhibition* consists of features that are also implicated in the core symptoms of ADHD. These features are conceptualized in ADHD as difficulties in executive functions or self-regulatory processes [71, 72]. Barkley [73] proposed that *behavioral inhibition*, consisting of: a) inhibition of pre-potent responses, b) stopping ongoing responses, and c) interference control, can be seen as a superordinate factor that affects several cognitive and behavioral modalities, eventually manifesting themselves in specific symptoms commonly found in ADHD. This construct is largely commensurate with Conscientious Inhibition as conceptualized in trait measures, thus providing a bridge between neuropsychiatric and quantitative trait approaches to conceptualizing underlying deficits in ADHD [14, 48, 74].

*Negative Emotionality* is elevated across many psychiatric disorders and is highly predictive of psychological and physical comorbidity as well as general quality of life [23, 48]. Although symptoms of Negative Emotionality are not central to ADHD criteria, this review and other evidence clearly shows that emotional distress and dysregulation are common among individuals with ADHD diagnoses [75]. There is also evidence that high Negative Emotionality also predicts worse outcomes in individuals with ADHD [76–78].

Significant variability was observed in effect sizes between and within samples in the current review, as well as between instruments. For example, the high percentage in the I^2^ measure, described above, indicates that there is a high degree of heterogeneity between studied populations within the different personality domains in the meta-analysis. Ideally, subgroup or moderator analyses would be performed to explain this variation. However, the number of studies available for this meta-analysis was too small to test moderation. Nevertheless, some potentially reliable patterns were observed.

First, when specific measures of FFM were extracted for separate analysis, heterogeneity decreased. This suggests more consistency when personality traits were measured with FFM instruments. One particularly important source of variability may have to do with differing facet models across those instruments. Facet variability is complicated by the interstitial nature of some traits, which leads different models to place similar facets on different domains. For instance, we conceptualized TCI Self-transcendence as an aspect of Negative Emotionality, although it also shares some features with Conscientious Inhibition. Future work exploring a broader range of traits, and conceptualizing personality at the level of facets, would shed significant light on this issue.

Second, stronger effects with psychopathology can generally be expected with maladaptive as opposed to normal range personality measures [16]. As maladaptive range measures are more likely to be used in clinical settings, associations with these measures are potentially both stronger, and more informative for clinical practice. As such, future research examining links between adult ADHD and maladaptive range personality measures would be particularly useful.

A third source of variability involves ADHD presentation, given that traits may differentially relate to inattentive, impulsive and hyperactive features [17, 45, 47, 51, 58]. Unfortunately, our relatively small sample size precluded moderator analyses of ADHD subtypes. Future studies with larger samples and symptom or subtype-level assessments of ADHD are needed to further explore the heterogeneity of effects we observed here.

### Clinical implications

Overall, these findings support the emerging view that personality, personality disorders, and neurodevelopmental disorders can be conceptualized within an integrated and evidence-based dimensional trait system. This supports the clinical application of personality trait assessment models according to the DSM and ICD diagnostic systems to broaden the clinical description of adult ADHD, as well as research on the shared etiological factors between personality variables and ADHD.

Categorical psychiatric diagnoses are fraught with high comorbidity, complicating the clinical picture and reducing treatment specificity. Dimensional models provide an opportunity to integrate comorbidity into a more comprehensive and individualized clinical description, which makes treatment planning more in line with current clinical praxis. The current review suggests that the features of ADHD can be economically situated within evidence-based models of personality and psychopathology. As such, the clinical use of personality trait measures and the emerging dimensional personality models included in the AMPD and the ICD-11 proposal provide opportunities to implement a more individualized approach for patients with problems related to hyperactivity, impulsivity, and inattention. Specifically, multidimensional trait profiles may help clinicians estimate long-term risk for psychosocial consequences of ADHD, as well as the likelihood of other comorbid conditions. They also help provide explanations for heterogeneous clinical presentations and point to appropriate pathways for intervention [41–43, 79].

In clinical practice it is useful to rely on heuristics to make efficient clinical decisions [80]. For example, these findings indicate that individuals with scores on personality measures that are more than one standard deviation higher on Negative Emotionality (NE) constructs or lower on Conscientious Inhibition (CI), in relation to non-clinical populations, may be at risk for ADHD or other disorders. Such heuristics can be helpful in screening because they both indicate risk for specific kinds of problems, while also efficiently providing a comprehensive portrait of the individual’s strengths and weakness, which has significant value for making prognostic predictions and guiding treatment [17, 43–45].

#### Limitations

The literature on associations between adult ADHD and personality traits is scarce, thus limiting the degree to which the current results can provide robust information about these links. The existing literature, while sufficient to quantitatively summarize connections between adult ADHD and IFFM trait domains, limited our ability to test moderators such as ADHD subtype, personality measure, or various sample features, and it necessitated inclusion of measures with varying connection to the FFM or DSM/ICD personality disorder proposals. Some of our conclusions were based on assumptions about how to organize facets into maladaptive five factor domain [37, 47] that have not been fully tested empirically. All personality measures were self-report. This is particularly important because some aspects of personality (developmental, variability, neurocognitive, reflective functioning) may be better assessed using other methods (e.g., informant reports, repeated reporting, implicit tests, neuropsychological testing). Also, the initial screening was performed only by the primary author, which might have risked possible exclusion of relevant studies. Subsequent multiple rater inclusion process was based on consensus; however, reliability statistics for how consensus was reached, were not calculated. Finally, the samples used in this study were WEIRD (i.e. *White Educated Industrialized Rich and Democratic*). Greater diversity in samples is needed to add confidence to these findings and enhance generalizability.

## Conclusion

Psychiatric nosology is in a transition from legacy diagnostic categories to evidence-based models of individual differences that closely resemble personality traits [14, 81]. Research on how ADHD fits into these models is relatively nascent, particularly in adult samples. This study shows that existing research on personality associations with ADHD suggests that ADHD is strongly related to Negative Emotionality and low Conscientious Inhibition, and moderately related to low Positive Emotionality and low Agreeable Inhibition. These results support the clinical assessment of personality traits in ADHD diagnosis, clinical care, and research, while also pointing to the need for further research to more specifically delineate how ADHD can be fit into the personality/psychopathology hierarchy.

## Data Availability

The datasets used and/or analysed during the current study are available from the corresponding author on reasonable request.
